# What Is Influencer Marketing and How Does It Target Children? A Review and Direction for Future Research

**DOI:** 10.3389/fpsyg.2019.02685

**Published:** 2019-12-03

**Authors:** Marijke De Veirman, Liselot Hudders, Michelle R. Nelson

**Affiliations:** ^1^Department of Communication Sciences, Ghent University, Ghent, Belgium; ^2^Department of Marketing, Ghent University, Ghent, Belgium; ^3^Department of Advertising, University of Illinois at Urbana–Champaign, Urbana, IL, United States

**Keywords:** social media influencers, influencer marketing, children, native advertising, advertising literacy, YouTube, vlogs

## Abstract

Children nowadays spend many hours online watching YouTube videos in which their favorite vloggers are playing games, unboxing toys, reviewing products, making jokes or just going about their daily activities. These vloggers regularly post attractive and entertaining content in the hope of building a large follower base. Although many of these vloggers are adults, the number of child vloggers is flourishing. The famous child vlogger Ryan of Ryan’s World, for instance, has more than 19 million viewers and he is (at age seven) a social media influencer. The popularity of these vloggers incited advertisers to include them as a new marketing communication tool, also referred to as influencer marketing, in their marketing strategy. Accordingly, many influential vloggers now receive free products from brands in return for a mention in one of their videos and their other social media (e.g., TikTok or Instagram) and some are even paid to create a sponsored post or video and distribute it to their followers. This sponsored content appears to be highly influential and may affect young children’s brand preferences. Given the limited advertising literacy skills (i.e., knowledge of advertising and skills to critically reflect on this advertising) of children under age 12, they are a vulnerable target group when it comes to persuasion. Therefore, caution is needed when implementing this marketing tactic to target them. However, research on how influencer marketing affects young children (under 12) is scarce and it is unclear how these young children can be empowered to critically cope with this fairly new form of persuasion. This paper therefore aims to shed light on why and how social media influencers have persuasive power over their young followers. The paper starts with providing insights into how and why social media influencers became a new source in advertising. We then discuss the few studies that have been conducted on influencer marketing among young children (under 12), based on a systematic literature review, and take these findings to formulate societal and policy implications and develop a future research agenda.

## Introduction

Today, children have plenty of options for digital entertainment and social media. YouTube in particular, has emerged as a platform for children’s screen time and an alternative for traditional television (TV) content (Watson, 2019). As an illustration, 81% of U.S. parents let their children under 11 watch YouTube (Pew Research Center, 2018), where they are exposed to advertising before they watch a video, and increasingly, brands are found within the videos, too (Weiss, 2018). YouTube offers new possibilities for brands to engage with children and their parents, including embedded advertising formats containing subtle brand integrations in entertaining media content, making them less intrusive, and thus harder to recognize (Hudders et al., 2017). For example, Ryan’s World^1^, which features a young boy reviewing branded toys and products, was the 6th most watched YouTube site for children, with more than 19 million viewers (April 2019; Clement, 2019). Given the popularity and potential to reach young viewers online, digital advertising spending for children reached 900 million U.S. dollars in 2018 and is forecast to increase in the future (Guttmann, 2019).

The stars of social media, also referred to as vloggers on YouTube, have become important influencers for the consumption decisions of their young audiences. They give their followers an insight into the brands they love and use in their daily life and even give direct advice on the products their followers should use or not use (De Jans et al., 2019). Because of their reach and the credibility they exude, many brands have added these influencers, who are often kids themselves (e.g., Stella and Blaise, The McClure Twins, AnnieLeblanc, or EvanTubeHD), to their marketing strategies. Although these endorsers may appear to be “ordinary” children, some are actually highly paid endorsers, for example, Ryan earning $22 million in 2018 (Robehmed and Berg, 2018). This is because, in return for free promotional goods or payment, brands ask these influencers to endorse their products on their social media profiles (on their feed or in their stories on Instagram, videos on YouTube and TikTok, or Facebook updates, etc.) and their YouTube channels in turn earn advertising revenue as a result of their large audiences. This is a phenomenon which has been referred to as influencer marketing (De Veirman et al., 2017). This form of marketing – the influencers’ social media contributions – is regarded as a form of advertising when (1) influencers receive a compensation (free products or financial payment) and (2) advertisers have control over the content, which also includes simple final approval of the post or general instructions regarding the post (e.g., I want two posts about our product). This definition of influencer marketing can be found in the guidelines of some advertising self-regulatory bodies (e.g., EASA, 2018).

Brands appear within influencers’ content as seamless implied endorsements. Hence, the branded content or advertising is fully integrated in the media content children are consuming (Hudders et al., 2017; De Jans et al., 2019). Moreover, despite clear guidelines from governmental regulators for mandatory disclosures of these endorsements (e.g., Federal Trade Commission in the U.S.), influencers often do not appropriately disclose the commercial nature of their posts (Children’s Advertising Review Unit, 2017; De Veirman et al., 2019). Ryan’s World, for example, was recently accused of deceiving children through “sponsored videos that often have the look and feel of organic content” in an official complaint by the watchdog group Truth in Advertising, filed with the Federal Trade Commission (Hsu, 2019). As a result, children (Folkvord et al., 2019) and even parents (Evans et al., 2018) may not understand that the YouTube content they are viewing is, essentially, an advertisement. However, despite their increasing prevalence and importance in children’s commercial media environment, research on how influencer marketing affects young children is still limited (for an exception, see De Jans et al., 2019). Most of the studies on influencer marketing focus on an adult audience and examine how influencers affect the purchase decisions of their followers (e.g., De Veirman et al., 2017; Lou and Yuan, 2019; Schouten et al., 2019).

Therefore, this paper aims to theoretically outlay how social media influencers, as a new source in advertising, target and affect young children. Children have always been an important target group for marketers, both because of their impact on their parents’ buying decisions, but also as future adult consumers (Calvert, 2018). We focus on children under age 12 as they are highly vulnerable to advertising. Their advertising literacy, all knowledge and skills related to advertising, is not yet fully developed (Hudders et al., 2017). The cognitive abilities, emotion regulation, and moral development are still immature for children under 12 (John, 1999; Rozendaal et al., 2011; Hudders et al., 2017). These abilities help them to understand the persuasive intent of advertising and strategies used to persuade them, control the emotions that advertisements may arouse, and evaluate the fairness and appropriateness of advertising (e.g., use of stereotypes). A strongly developed advertising literacy is indispensable to be able to critically reflect on advertising and avoid subconscious persuasion (Hudders et al., 2017). Not surprisingly, several studies have shown that children under 12 have difficulties to cope with embedded advertising (e.g., Hudders et al., 2016; De Pauw et al., 2018a,b). Accordingly, specific corporate actions and self-regulations apply for children under 12 in order to protect them from advertising influence. For instance, several European food brands agreed not to advertise unhealthy food products to children under 12 in the EU Pledge^2^ agreement as they are aware of children’s high susceptibility for their advertising messages. Since 2019, influencer marketing has been included in this agreement.

The purpose of this review is threefold. First, the research aims to provide insights into the importance of social media influencers as a source in advertising targeting children. In order to adopt a theoretical approach to children’s processing of influencer marketing, we draw upon existing theoretical and empirical work regarding source effects in persuasion. During childhood, children encounter different advertising sources shaping their tastes and preferences, trying to turn them into brand loyalists as they grow older. Social media influencers can be perceived as a new type of real-life endorser affecting children’s and their parents’ consumption behavior. Second, this paper provides a review of the current (limited) academic research on influencer marketing targeted at children. Additionally, societal and policy implications of this tactic in the context of prior research are discussed. Third, a future research agenda that may foster academic research on the topic is included. This way, we hope our review may provide a basis for marketing-related regulation, policies, and parent intervention strategies and thus help ensure the protection of children.

## Social Media Influencers as a New Source in Advertising Targeting Children

### Source Effects in Persuasion

The importance of the source as sender of the object of information has been well recognized in the communication and advertising literature. Already in 1948, Laswell emphasized that every communication involves a message, a medium, a recipient, and a source (Lasswell, 1948). In advertising, a distinction can be made between the one who bears financial and legal responsibility for the message and the one who communicates the brand’s message (Stern, 1994). As such, in the latter sense, brands may create or license a character or pay an endorser or spokesperson to influence consumers’ attitudes and purchase intentions. The persuasiveness of advertising is strongly influenced by consumers’ perception of these sources (see Wilson and Sherrell, 1993 for a meta-analysis). For instance, source credibility plays a critical role in how consumers evaluate brands and products, whereby a positive evaluation of the credibility of the source is likely to translate into positive advertising outcomes (Sternthal et al., 1978; Ohanian, 1991). *Source credibility* consists of two dimensions: trustworthiness and expertise. Trustworthiness refers to the honesty, believability, and morality of the endorser, while expertise refers to the endorser’s competence, knowledge, and skills (Hovland et al., 1953; Sternthal et al., 1978; Erdogan, 1999; Flanagin and Metzger, 2007). Both trustworthiness and expertise have been found to enhance advertising effectiveness (Amos et al., 2008). Furthermore, the physical appearance or *attractiveness* of the source plays a major role in the endorsers’ credibility and, consequently, persuasiveness (Kahle and Homer, 1985; Kamins, 1989; McCracken, 1989; Ohanian, 1991). Even among children, using attractive peer models has been shown to increase advertising effectiveness (e.g., Van de Sompel and Vermeir, 2016). Source attractiveness is driven by factors such as perceived similarity, familiarity, and likeability of the source. Familiarity refers to the extent to which one knows the source through exposure; likability is defined as affection for the source as a result of the source’s physical appearance and behavior, while similarity is the supposed resemblance between the source and receiver of the message (McGuire, 1985). According to McGuire (1985), sources who are known to, liked by, and/ or similar to the consumer are found to be attractive and, as a result, are persuasive. Moreover, these source factors contribute to consumers’ *identification* with the source, which increases the likelihood they will adopt their beliefs, attitudes, and behaviors (Kelman, 1961; Basil, 1996).

When consumers identify with the source, they will likely imitate his/her behavior, including the products he/she uses (Kelman, 1961), also referred to as *social learning* (Bandura et al., 1961). Indeed, as sources in advertising usually show the usefulness of the product they endorse and how to use it, this action may lead to observational learning and modeling of their behavior accordingly (Bandura et al., 1966). Moreover, next to family and friends, sources used in advertising may serve as role models they refer to in their identity formation (Lloyd, 2002; Hoffner and Buchanan, 2005). As a result, when advertising sources are paired with products, the positive affective responses toward those sources may transfer onto the products (Acuff and Reiher, 1999; McNeal, 2007; de Droog et al., 2012), which has been referred to as a *meaning transfer* (McCracken, 1989). Importantly, crucial to the effectiveness of endorsement marketing is a good fit between the source and the product he/she endorses, which has been referred to as the match-up hypothesis in celebrity research (Kamins, 1990). To conclude, the likelihood of the former process to occur increases when consumers develop a *parasocial relationship* (parasocial interaction; PSI) with the source (Tsay-Vogel and Schwartz, 2014). PSI, as introduced by Horton and Wohl (1956), refers to the relationships consumers develop with media characters, making them important sources of information (Rubin et al., 1985). Accordingly, when consumers identify with a source and their brand-usage behavior, they will likely adopt this behavior (Naderer et al., 2018). As the need for companionship, which is the main driver for relationship formation, emerges in childhood (Hoffner, 2008), children typically engage in the formation of PSI’s (de Droog et al., 2012).

The above theoretical insights explain the success of sources used in advertising in general. As such, this robust body of literature has demonstrated that the source in the message is important for persuasion effects. These processes may also apply to social media influencers. Before discussing how influencers may affect children, we will first provide an overview of the different sources that are used in advertising targeting children and discuss how influencers evolved as a new source in advertising.

### Insights Into the Persuasiveness of Different Types of Sources Targeting Children

#### Brand and Licensed Characters

Brands often display anthropomorphized or fantasy characters in their advertising and on their product packaging. These cartoon-like figures can be created by the brand with the sole purpose of promoting their products and services (i.e., brand mascots, brand icons, and non-celebrity spokes-characters). Famous examples of brand mascots are Tony the Tiger (Kellogg’s Frosted Flakes breakfast cereal), Chester Cheetah (Frito-Lay’s Cheetos), Ronald McDonald (McDonalds), and Mr. Peanut (Planters). These characters are in full ownership of the brand, which implies a very high level of control over the source and its message. However, as it takes a lot of development and marketing efforts to build awareness and affinity for these characters, brands may also opt to license a character from media companies [i.e., licensed (media) characters, also called celebrity spokes-character] (Kraak and Story, 2015). Examples of licensed characters that are used by brands are numerous. For instance, Spongebob Squarepants endorses Yoplait GoGurt and Disney’s Cars figures endorse Hot Wheels toys. McDonalds licensed different characters over time to endorse their happy meals (e.g., The Incredibles, My Little Pony, and Transformers Cyberverse). Spider Man endorses everything from Pop-Tarts to Cheez-it crackers to Fruit Snacks to Fudge Stripe cookies (for more examples, see licenseglobal.com). Contractual agreements allow them to use the characters for merchandizing and cross-promotions (Kraak and Story, 2015; Smits et al., 2015). As a result, popular media characters are highly present in children’s daily lives, not only through television and other media, but also through merchandising (e.g., books, toys, and clothes featuring characters; Atkinson et al., 2015) and on product packages and in advertising.

Brands profit from these characters’ fame and popularity among children. For instance, in the fourth quarter of 2018, U.S. consumers spent approximately 21.6 billion dollars on licensed merchandise products for children (O’Connell, 2019). Apart from the fact that they are effective advertising sources, brand and licensed characters hold the advantage that, since they are “fictional” (and therefore more “controllable”), they are less likely to be embroiled in a scandal as compared to real-life endorsers, which might negatively affect the brand image as negative associations people make with a celebrity can be transferred to the brand (e.g., Till and Shimp, 1998; Stafford et al., 2002). Furthermore, these characters do not age or change much over time, which makes their employability less timely as compared to human endorsers.

Research on the use of brand mascots and licensed characters on children’s attitudes and behaviors has mainly focused on food with findings suggesting that the presence of cartoon media characters positively affects children’s food preferences, choices, and intake, especially for energy-dense and nutrient-poor foods as compared to fruits or vegetables (see Kraak and Story, 2015 for a review). The presence of brand mascots and licensed characters draws children’s attention (Ogle et al., 2017), helps them recognize and recall the products and brands they endorse, and has been found effective in improving attitudes about the advertised product (e.g., Neeley and Schumann, 2004; Lapierre et al., 2011) and increasing product choice (e.g., Nelson et al., 2015).

There are multiple reasons why these sorts of sources are attractive for children. First, these simply drawn, colorful and funny characters appeal to them and are easy to remember, which is helpful given the limited cognitive abilities of young children (de Droog et al., 2011; Lapierre et al., 2011). Second, children easily form parasocial relationships with these likeable and fun characters (de Droog et al., 2012), which may result in a preference for the products they endorse (Lagomarsino and Suggs, 2018). Moreover, as these characters are now finding their way onto social media (e.g., Chester Cheetah has 191k followers on Instagram), the perceived distance between (child) consumers and their favorite characters is reduced, which may even increase feelings of parasocial interaction. Third, the pairing of brands with popular cartoon media characters is not only limited to packaging and traditional advertising, but also takes on more integrated forms. For instance, likeable characters may interact with and consume branded products in children’s movies (Naderer et al., 2018, 2019), or brands may be integrated in highly interactive and engaging (adver)games featuring likeable and even customizable avatars (Bailey et al., 2009). As these placements are embedded in the media content, children may experience extra difficulties recognizing the persuasive intent of these placements (see De Jans et al., 2019c for an overview).

Given their persuasiveness, European food brands have agreed not to use licensed characters (e.g., Spongebob Squarepants and Spiderman) to promote unhealthy food products to children under 12 as a part of the European Pledge agreement (EU Pledge, 2019). Company-owned, brand mascots (e.g., Tony the Tiger and Chester Cheetah) do not fall under this agreement; however, implying that brand characters can still be widely used to endorse unhealthy food products.

#### Real-Life Endorsers: Celebrity and Peer Endorsers

Next to highly engaging and fun characters, real-life endorsers are also frequently used to mold children’s consumption. For instance, brands may tie (child) celebrities to their products and use them as endorsers. Children look up to these publicly recognized figures who may serve as role-models to them (Read, 2011; Power and Smith, 2017). Brands aim for an image-transfer, hoping that positive associations attached to celebrities will transfer to the brands those celebrities endorse (McCracken, 1989), and children will wish to identify with them as they aspire to be like the celebrity (Kamins et al., 1989). Celebrity endorsement as an advertising strategy has been widely researched among adult populations (see Knoll and Matthes, 2017 for a review), but has also been found a valuable strategy to target children as it may impact their attitudes, preferences, and behaviors (e.g., Ross et al., 1984; Jain et al., 2011). Research on the impact of celebrity endorsement on children has mainly focused on food marketing and has found that celebrities and athletes who are popular among minors typically endorse energy-dense and nutrient-poor products (e.g., Bragg et al., 2013, 2016), which may increase children’s intake of these less-healthy foods (e.g., Boyland et al., 2013; Dixon et al., 2014). On the other hand, despite their presumed lower advertising literacy, children do have a fairly good understanding of celebrity endorsement, which they develop at an earlier age compared to their understanding of other advertising techniques (Rozendaal et al., 2011).

As an alternative to using celebrities as endorsers, advertising aimed at children typically presents (unknown) actors and models interacting with or consuming branded products. For instance, a recent content analysis of 506 food and beverage advertisements aired on the Nickelodeon channel found that most of the ad endorsers were “general people,” either alone (*N* = 232, 45.8%) or paired with brand mascots and licensed characters (*N* = 82, 16.2%), while advertisements with celebrity endorsers accounted for 7.5% (*N* = 38) (Ahn et al., 2019). Next to adults, these actors typically appear as “regular” children and teenagers with whom their young audience can easily identify. Accordingly, the purpose of these ads is to evoke peer modeling or social learning, which implies that children will copy the peer’s behavior, including their consumption patterns (Bandura, 2002). Indeed, gaining peer acceptance and approval is important to children (Mangleburg et al., 2004; Devlin et al., 2007). Particularly from the age of 6–7 years, peers become important agents for consumer socialization and children start modeling peers’ behavior (John, 1999). Even pre-school children’s behaviors and consumption may already be influenced by their friends (Ahn and Nelson, 2015; Atkinson et al., 2015). This has shown to be particularly effective when the peer is of the same age or slightly older than themselves (Brody and Stoneman, 1981). Moreover, children are more likely to engage in peer modeling, when the peer’s behavior is being rewarded, which is mostly the case in advertising (Flanders, 1968). For instance, a content analysis of food marketing targeted at children found that these children are typically depicted as happy, playing with friends and enjoying the foods offered to them, thus conveying the indirect message that children will be happier when they consume these foods (Hebden et al., 2011).

The rise of social media introduced a new type of peer endorsement, namely social media influencers. In the following section, we will describe the rise of these social media influencers as a new source in advertising to target young children.

### Conceptualizing Social Media Influencers as a New Advertising Source

#### The Emergence of Social Media Influencers as Credible Advertising Sources

The proliferation of social network sites has extended the reach of word-of-mouth and amplified the impact of peer recommendations, which can now be shared one-to-many (Lyons and Henderson, 2005; Boyd and Ellison, 2007; Knoll, 2016). Children today are audiences and creators of media. Children can easily generate electronic word of mouth (eWOM) themselves, which increases the brand’s visibility and awareness and has been shown to positively affect children’s products’ sales (Bao et al., 2019). For instance, children are able to share online reviews (e.g., Amazon enables children under 13 to upload their own reviews which are marked as “A kid’s review”), engage with brands on social media (e.g., despite the age-limit of 13 years, children can easily create a Facebook or Instagram account with false information), and create and share their own videos, including showing their ownership of and experiences with products (e.g., YouTube Kids allow children under 13 to create an own account). As peers are believed to have no commercial interest, they are perceived as authentic and credible sources of information. As a result, children and their parents may be less resistant toward their endorsements compared to brand-originated marketing communication and sources (de Vries et al., 2012; van Noort et al., 2012). Accordingly, brands aim to encourage and reward eWOM (e.g., free gift in return for a review or online check-in, like and share contests), resulting in (minor) consumers becoming advertising sources rather than merely targets (Chu and Kim, 2011). However, the high credibility and impact of these real-world endorsers may be offset by their uncontrollability (Yang, 2017).

Recognizing the impact of eWOM on consumers’ attitudes and decisions, brands have started approaching social media influencers (i.e., influential social media users who managed to build a large audience of followers or subscribers), and incentivizing them to create and distribute relevant, authentic-looking brand-related content, a practice that is called influencer marketing (De Veirman et al., 2017). This way, brands may leverage the power of word-of-mouth while re-gaining control as official agreements between brands and the influencers are made. Influencers will likely be positive as they wish to remain loyal toward the brands they receive incentives from (De Veirman et al., 2019).

Social influencers do not only attract minors’ attention, the child audiences also aspire to become influencers themselves (Chambers et al., 2018). Some children indeed managed to build a large audience of followers or subscribers on their media channels, which are often run by their parents (Novacic, 2019). They mostly maintain multiple social media accounts, with Instagram, TikTok, and YouTube as the most important ones on which they post entertaining and inspiring content (De Veirman et al., 2019). There are even a number of very young child influencers or “kidfluencers,” who post a variety of videos on YouTube, including toy reviews, fun challenges and tutorials [e.g., EvanTubeHD (age 13); Ryan’s World (age 7)], or who maintain a successful Instagram channel [e.g., Call Me Sparkle (age 5); Zoooey in the City (age 7)]. Different than, for instance, child actors, the influencer does not pretend to play and have fun with toys, he/she really does, increasing their relatability to the audience and blurring any persuasive or selling intent.

As a high number of followers and likes are likely to result in a wide reach of the (commercial) message, these remain key metrics for brands, influencer agencies, and platforms aiming to identify and select appropriate influencers for their campaigns. This unfortunately also contributes to malicious practices of influencers buying fake followers and likes, for instance, through bots and clickfarms, to artificially inflate their perceived influencer status. On the one hand, this may lead to disappointing results for brands as their influencer marketing campaigns do not achieve objectives. On the other hand, when the public finds out their favorite influencer bought fake followers and likes, they may think of him/her as a fraud, resulting in negative reactions and unfollowing (De Veirman et al., 2017). Moreover, influencers risk punishment, for instance, by the FTC who has the authority to bring enforcement actions against deceptive or unfair marketing practices (Section 5 of the FTC Act). Recently (October 2019), the FTC halted deceptive online marketing practices related to influencer marketing. The regulatory body showed evidence of a company (Devumi) that sold fake accounts and indicators of social engagement and influence to various influencers. Specifically, the FTC complaint suggests that the company: “Devumi filled more than 58,000 orders for fake Twitter followers, enabling the buyers to deceive potential clients about their social media influence. The complaint cites over 4,000 sales of fake YouTube subscribers and over 32,000 sales of fake YouTube views.” (Fair, 2019b). The option to buy fake followers and likes through a single click is particularly worrying when it comes to minors, both because of the financial burden and the legal consequences they risk.

#### Social Media Influencers as Relatable Role Models

Similar to traditional celebrities, influencers with a lot of followers on one or more platforms (e.g., so-called macro influencers have between 100,000 and 1 million followers; Ismail, 2018) have a large reach; however, the origin of their fame and nature of their influence is different. While traditional celebrities gained public recognition because of their extraordinary beauty (e.g., supermodels) or talent (e.g., actors and athletes), social media influencers branded themselves on social media through posting highly appealing self-generated content, either on a specific topic in which they present themselves as an expert (e.g., food, beauty…), or more generally, showcasing their lifestyles as a whole (Khamis et al., 2017; De Jans et al., 2019). Children merely watch influencers’ videos for entertainment purposes. However, the content can also become informative for children’s consumption decisions as the influencers often let them know which brands they like and dislike and overtly display these brands in their videos (Martínez and Olsson, 2019). Different than traditional celebrities, who are nowadays also present on social media and extended their fame online, social media influencers have built their fame on social media, without being known to the public beforehand (De Veirman et al., 2017; Schouten et al., 2019). A distinction can be made between different types of influencers, depending on their number of followers. As such, social media influencers may start out with a small audience of engaged followers (less than 1,000) with whom they have a real-life relationship, resulting in high perceptions of authenticity. As they profile themselves as opinion leader in a specific niche, resulting in a growing audience, they are referred to as micro-influencers (1,000–100,000 followers). Some influencers even manage to build very large audiences (1,000,000–1 million followers) on social media and gain celebrity status (Ismail, 2018; Alassani and Göretz, 2019).

Children look up to (child) influencers, but at the same time, they believe they are highly similar to them as they share the same interests and activities and they seem to be ordinary children just like them (van Dam and van Reijmersdal, 2019). Despite their online celebrity-status, influencers tend to be perceived as very relatable and approachable, due to the highly personal content they post and the interactions they have with their followers. Their social media posts provide their followers a glimpse into their personal lives, resulting in perceptions that they have a lot in common with the influencer and he/she is a peer (Abidin, 2015; Gannon and Prothero, 2018; Schouten et al., 2019). Therefore, children are likely to build a relationship with their favorite influencers, which can be referred to as parasocial interaction (cfr. supra), which implies that they become influential sources of information and inspiration that may impact consumption-related decisions (Rubin et al., 1985; De Jans et al., 2019). Lee and Watkins (2016) for instance showed that developing parasocial relationships with a vlogger may positively affect brand perceptions, and De Jans et al. (2019) found it to be positively related with purchase intentions. Moreover, as influencers show the usefulness of the products they endorse, children may learn and model their behavior (Akers et al., 1979; Lagomarsino and Suggs, 2018), which has been referred to as peer modeling or social learning, which can be particularly effective for persuasion when the child likes or admires the model (Bandura, 1977). For instance, Ryan (of Ryan’s World), today’s most popular child influencer with 21 million subscribers to the YouTube channel, shows his daily routines, including the type of cereal he eats, the toys he plays with, and the places he goes to in his vlogs. Accordingly, it is likely that children will perceive child influencers such as Ryan as peers, who are an important source of influence in their consumer socialization process (Bachmann et al., 1993). As a result, children may be very willing to identify with popular child influencers and take on their attitudes, beliefs, and behaviors, including those related to brands and products (Kelman, 2006; Pilgrim and Bohnet-Joschko, 2019; Schouten et al., 2019).

#### Social Media Influencers as Hidden Persuaders

Influencers have become an important asset for advertisers, as they directly address their target audience and include brand and product recommendations in highly relevant and entertaining content. The practice of “influencer marketing” refers to advertisers closing deals with influencers, which entail promotion in exchange for payment, free products or invitations to exclusive events (De Veirman et al., 2017). Doing so, advertisers wish to create a meaning transfer (Russell, 1998) and reach their target audience in an authentic way. Influencers usually hold a lot of creative freedom in content creation, as they know their audience best. They incorporate branded content in reviews, recommendations, and tutorials in real-life settings, which increases their trustworthiness as they have tried the products themselves and promote them in an authentic manner (Uzunoglu and Kip, 2014; Schouten et al., 2019). Influencers’ so-called sponsored posts or videos mimic and blend with organic content in users’ feeds without disturbing their mindless scrolling of social media (Abidin, 2016; Wojdynski and Evans, 2016; De Veirman et al., 2017). Accordingly, the practice of influencer marketing can be considered a form of native advertising, defined as “any paid advertising that takes the specific form and appearance of editorial content from the publisher itself” (Wojdynski and Evans, 2016). Moreover, although today there are clear guidelines and regulations on disclosing sponsored content, influencers are hesitant to transparently disclose the commercial nature of their posts, either because they are not aware of the rules or because they want to avoid irritation among their followers (De Veirman et al., 2019).

Therefore, children may fail to recognize influencer content as advertising and cope with such persuasion tactics critically (Friestad and Wright, 1994; De Veirman et al., 2019). As a result, instead of advertising, influencers’ brand-initiated endorsements are likely to be perceived as highly credible electronic word-of-mouth the influencer shares out of genuine liking of the brand (Phelps et al., 2004; Cheung et al., 2009). Moreover, due to the correspondence bias, people (and children in particular) tend to believe that although the influencer was compensated for endorsing a brand, he/she would not do this if he/she would not truly like the brand. This bias is the tendency to believe that a person’s behavior is a true reflection of their true underlying dispositions when in fact, their behavior could be explained by situational factors, i.e., the influencer was compensated by the brand (Gilbert and Malone, 1995; O’Sullivan, 2003). Interviews with teenage influencers recently revealed that they indeed only wish to endorse brands they fully support; however, when dissatisfied with a received product, a dilemma between being truthful toward their followers and being loyal to the brand arises (De Veirman et al., 2019).

Children are extra vulnerable when it comes to this type of native advertising, as their advertising literacy is not fully developed yet. Advertising literacy refers to an individual’s knowledge of and skills related to advertising (i.e., dispositional advertising literacy) and to their ability to recognize and critically evaluate it (i.e., situational advertising literacy) (Friestad and Wright, 1994; Hudders et al., 2017). The embedded nature of influencer marketing lowers both children’s ability and motivation to recognize it as advertising and critically reflect on it (Nairn and Fine, 2008). Their ability is lowered as influencers’ sponsored posts and videos appear between non-sponsored content in their feeds, which results in the simultaneous exposure of editorial and commercial messages. This content overload makes it hard for children to focus their attention and discern relevant from irrelevant information, resulting in a depletion of self-regulatory resources and difficulties to critically reflect on commercial messages. Second, their motivation is lowered as the commercial messages are integrated in fun and engaging posts and videos (Hudders et al., 2017). Remarkably, influencers’ videos are available on YouTube Kids (e.g., Ryan’s World, today’s most popular child influencer channel), which parents perceive as a safe media environment. Indeed, while YouTube states that the limited ads on YouTube Kids undergo a rigorous review process for compliance with their policies (YouTube, 2019a), these covert advertising practices slip through the net.

### Current Knowledge on Influencer Marketing Targeting Children

#### Selection of Empirical Studies on Influencer Marketing Focusing Among a Child Sample

A literature search was done employing a “title-abstract-keywords” search in the Scopus database using the keywords (influencer OR blog* OR vlog* OR microcelebrit* OR unboxing) AND (child* OR kid* OR minor* OR youth) AND (marketing OR advertis* OR commercial*) to get an insight into the literature that specifically focuses on social media influencers as an advertising source targeting children. At the time of our research (August 2019), this search process yielded 55 academic articles. The papers were screened to exclude those not relevant to the limited research overview. Papers were only included if they met the following predefined criteria: (1) only peer-reviewed articles, (2) articles examining children up to 12 years old, and (3) articles published in English. All articles were inspected by reading titles and abstracts to determine if they met the proposed criteria. After screening, seven academic articles that matched our criteria were found. However, we also decided to include one article (Evans et al., 2018), which examined young children’s parents, rather than children directly. Furthermore, we checked the lists of “latest articles” of 25 SSCI-ranked journals from the domains of communication, media, marketing, and advertising, including two journals that specifically focus on children (see Table 1). No additional articles that comply with the inclusion criteria were detected. Below, we will briefly discuss the eight included articles. Given the small sample, we briefly summarize each of the articles below and then we compare and synthesize what is known about influencers and children in this nascent body of literature.

**Table 1:** List of 25 selected SSCI-ranked journals.

New Media & Society Journal of Communication Journal of Computer-Mediated Communication International Journal of Communication Communication Research Social Media + Society Media, Culture & Society Communication Theory European Journal of Marketing International Journal of Advertising International Journal of Research in Marketing Journal of Advertising Journal of Advertising Research Journal of Interactive Marketing Journal of Marketing Journal of Marketing Research Journal of Public Policy & Marketing Journal of the Academy of Marketing Science Marketing Letters Marketing Science Psychology & Marketing Cyberpsychology Cyberpsychology, Behavior & Social Networking Young Consumers Journal of Children & Media

#### Overview of Empirical Studies

Eight empirical studies examined the practice of influencer marketing among a young audience (see Table 2). One study reports the results of a content analysis of branded vlogs and three studies (one survey study, one focus group study, and one ethnographic study) focused on how children think about this sponsored vlogging. Three studies experimentally examined the effectiveness of sponsored vlogging, with two studies focusing on its effect on children’s food intake and one study focusing on the effect of an educational vlog on children’s susceptibility to vlog advertising. To conclude, one study examined how parents cope with sponsored vlogs. Remarkably, all studies focus on YouTube as the main channel for influencer marketing. Below, we discuss the studies in more detail.

**Table 2:** List of the eight included articles.

Authors	Year	Title	Journal
Nicoll and Nansen	2018	Mimetic production in YouTube toy unboxing videos	Social Media + Society
Marsh	2016	“Unboxing” videos: co-construction of the child as cyberflâneur	Discourse: Studies in the Cultural Politics of Education
Martínez and Olsson	2019	Making sense of YouTubers: how Swedish children construct and negotiate the YouTuber Misslisibell as a girl celebrity	Journal of Children and Media
Folkvord, Bevelander, Rozendaal, and Hermans	2019	Children’s bonding with popular YouTube vloggers and their attitudes toward brand and product endorsements in vlogs: an explorative study	Young Consumers
Coates, Hardman, Halford, Christiansen, and Boyland	2019	Social media influencer marketing and children’s food intake: a randomized trial	Pediatrics
Coates, Hardman, Halford, Christiansen, and Boyland	2019	The effect of influencer marketing of food and a “protective” advertising disclosure on children’s food intake	Pediatric Obesity
De Jans, Cauberghe, and Hudders	2019	How an advertising disclosure alerts young adolescents to sponsored vlogs: the moderating role of a peer-based advertising literacy intervention through an informational vlog	Journal of Advertising
Evans, Hoy, and Childers	2018	Parenting “YouTube natives”: the impact of pre-roll advertising and text disclosures on parental responses to sponsored child influencer videos	Journal of Advertising

##### What Content Is Featured in Sponsored Vlogs and how Is It Perceived by Children?

One study performed a quantitative content analysis of branded vlogs. Nicoll and Nansen (2018) examined the content of 100 recent toy unboxing videos. They collected the videos by applying the “most recent” filter to the search term “toy unboxing” and analyzed the first 50 results on 2 days. They made a comparison between the vlogs of children (53%) and adults (47%) and analyzed variations of expertise, professionalism, and promotion across the vlogs. The age of the child unboxers ranged from toddler (2–4 years; 9%) to adolescent (12–18 years; 5%), with the majority (39%) being primary school age (4–11 years). Concerning their gender, 36% of endorsers were female and 52% male, while gender was undecided in 11% of the videos (e.g., only hands were visible, no commentary). Boys mostly unboxed (and assembled) toy cars and Legos, whereas girls tended to unbox toys like Shopkins (tiny collectable toys). Their main conclusion was that the toy unboxing videos of children tended to be much more varied and used more everyday language compared to professional (e.g., EvanTubeHD) or adult’s videos, yet they seek to mimic adult and professional videos, including their production and branding strategies. On the other hand, well-known professional channels try to produce a semblance of amateur authenticity by imitating the playful qualities of children’s videos (Nicoll and Nansen, 2018).

Next, three studies examined what children think about sponsored vlogs. Marsh (2016) took an ethnographic approach to observe how a 4-year-old child engages with unboxing videos on YouTube. According to this study, the young child took the position of cyberflȃneur who seemed to enjoy the mere act of viewing, while not engaging in nagging behavior, asking his parent to purchase the products.

Martínez and Olsson (2019) conducted a focus group study to examine how the practice of sponsored vlogging is perceived among 9- and 12-year old Swedish children where 12 focus groups with 46 children were conducted. Participants were shown a video (make-up tutorial) of the popular YouTuber Misslisibell as an illustrative example of advertising in vlogs. They had normative discussions around her vlogging practices and YouTube celebrity status related to her young age and had various interpretations of the video as advertising. The study shows the importance of YouTubers to children in their construction of identity and as a role model that guides their consumption (Martínez and Olsson, 2019).

The study of Folkvord et al. (2019) is an exploratory survey study among pre-teens/teens (10–13 years) in which they examined the amount of time children spend on viewing vlogs, their awareness and understanding of the endorsed brand in vlogs and their self-perceived susceptibility to the potential persuasive effects of these vlogs. Their conclusion was that the majority of children frequently watch vlogs and that their degree of bonding with the vlogger predicted the time they spend on watching vlogs. Moreover, they could easily recall products and brands displayed in the vlogs (mostly food and beverages) and believed that they themselves and others were affected by endorsements in vlogs.

##### Effectiveness of Influencer Marketing

Three experimental studies examined how children are affected by sponsored vlogs and one study focused on how parents of children between the ages of four and 11 react to sponsored vlogs. First, two studies examined the impact of social media influencer marketing on children’s food intake (Coates et al., 2019a,b). The first study is a randomized trial with a between-subjects design in which 176 children (9–11 years) were exposed to mock-Instagram profiles of two popular vloggers, either promoting healthy or unhealthy snacks. Results showed that influencer marketing of unhealthy foods increased children’s immediate food intake, whereas the equivalent marketing of healthy foods had no effect. In their second study, an advertising disclosure was included to alert children on the inclusion of advertising. In a between-subjects design, 151 children (9–11 years) were exposed to popular YouTubers’ vlogs including a non-food product, or an unhealthy snack with or without an advertising disclosure. Participants’ intake of the marketed snack and an alternative brand of the same snack were measured. In line with their first study, influencer marketing increased children’s immediate intake of the promoted snack relative to an alternative non-food brand (control condition). Remarkably, the inclusion of an advertising disclosure increased the effect, as children who viewed food marketing with a disclosure (and not those without) consumed 41% more of the promoted snack compared to the control group.

The study of De Jans et al. (2019) examined how an educational vlog can help children (11–14 years) cope with advertising. In an experimental study (*N* = 160), using a two (advertising disclosure: no disclosure versus disclosure) by two (peer-based advertising literacy intervention) between-subjects design, they examined how an advertising disclosure can reduce the persuasiveness of sponsored vlogs and how watching an educational vlog moderates these effects. The results show that an advertising disclosure increased their recognition of advertising and their affective advertising literacy for sponsored vlogs, and that only affective advertising literacy negatively affected influencer trustworthiness and PSI and purchase intention accordingly. Moreover, when young adolescents were informed about advertising through an informational vlog (i.e., peer-based advertising literacy intervention), positive effects on the influencer and subsequently on advertising effects were found of an advertising disclosure.

Evans et al. (2018) examined how parents of young children cope with sponsored vlogging on YouTube. They gauged parents’ understanding of and responses to sponsored child influencer unboxing videos. Through an experimental design among 418 parents, they assessed the influence of sponsorship text disclosure (present or absent) and sponsor pre-roll (sponsor pre-roll, nonsponsor pre-roll, and no pre-roll) for a toy on conceptual persuasion knowledge, perceptions of sponsorship transparency, and different outcome measures. Moreover, they explored how parental mediation influences the outcomes. They found that sponsor variations in pre-roll advertising (sponsor versus nonsponsor versus none) and sponsor text disclosure (present versus absent) conditions did not affect parents’ conceptual persuasion knowledge of the unboxing video. However, if a sponsor pre-roll ad was included, parents reported higher levels of sponsorship transparency of the unboxing video compared to parents who saw no pre-roll ad or a nonsponsor pre-roll ad. There was no additional effect on sponsorship transparency or conceptual persuasion knowledge when a sponsor text disclosure was included. Moreover, high levels of parental mediation conditionally impacted the indirect effect of a sponsor pre-roll advertisement *via* sponsorship transparency on perceptions of the unboxing video and attitudes toward the sponsor.

#### Conclusions

Four observations can be made based on this thorough review of academic literature. First, all of the included articles focus on YouTube as a platform, which is not surprising as it is frequently used among children (Pew Research Center, 2018; Ofcom, 2019). Only one study (Coates et al., 2019a) used mock-Instagram profiles in a randomized trial; however, these profiles represented popular YouTubers among children. No studies yet focused on TikTok, a social media platform which has become very popular among young children. Second, all the above studies except for one were conducted among teenagers with ages between 9 and 14 years old. Only one study (Marsh, 2016) focused on preschool children (4-year-old children) and no studies yet focused on children between 6 and 8 years when examining influencer marketing. Third, most articles take a consumer perspective and examine influencer practices and how minors perceive the persuasion tactic, while specifically focusing on persuasive content and perceptions of transparency, including the use of advertising disclosures, using different methods. Only one study conducted a content analysis of (child) influencers’ vlogs (Nicoll and Nansen, 2018). A final minor observation is the focus on (unhealthy) food products (Coates et al., 2019a,b), beverages (De Jans et al., 2019), and toys (Evans et al., 2018) in the experimental studies gauging for influencer marketing effects on children.

#### Implications

A number of societal and policy implications arise from the findings of prior research discussed above. More specifically, they may be important for policy aimed at protecting children’s interests. Most prior research focused on children’s exposure to and perception of influencer marketing. The above studies show that children spend a lot of time watching videos of their favorite influencers, in which they also encounter influencer marketing practices (e.g., Marsh, 2016; Folkvord et al., 2019; Martínez and Olsson, 2019). Moreover, they are also influenced by the content these influencers post and susceptible to the commercial messages incorporated in it (e.g., Folkvord et al., 2019; Martínez and Olsson, 2019), specifically those on (unhealthy) foods and beverages (e.g., De Jans et al., 2019; Coates et al., 2019a,b). Considering children’s susceptibility to influencer marketing practices, the findings of the above discussed prior research support the importance of existing guidelines and regulations stressing the fact that advertising should be recognizable as such, in particular for minor audiences. Two studies (De Jans et al., 2019; Coates et al., 2019b) tested the impact of an advertising disclosure on minors’ recognition of and susceptibility to influencer marketing. While both studies have shown that the inclusion of an advertising disclosure helps children recognize advertising, the study of Coates et al. (2019b) found that this increased recognition actually increased the effectiveness of influencer marketing, resulting in a higher intake of the promoted snack. Moreover, De Jans et al. (2019) found that the inclusion of an advertising disclosure may have positive effects on the influencer and subsequently on advertising effectiveness when minors were informed about advertising through an advertising literacy intervention in the format of an informational vlog. These studies suggest that a disclosure indeed helps children recognize influencer marketing practices as advertising and thus protects children from subconscious persuasion, without necessarily having a negative impact on the influencer and advertising effectiveness. In addition, Evans et al. (2018) found that sponsorship transparency in child-directed content is also appreciated by parents and has a positive effect on parents’ perceptions of unboxing videos, attitude toward the brand, and attitude toward the sponsor.

While attempts have been made, for instance in the EU (EASA) and the US (FTC), to formulate clear guidelines on the disclosure of influencer marketing, thus far an international, coordinated approach is lacking (De Jans et al., 2019). As a result of all these well-intended regulatory attempts, today there is a plethora of disclosures and wordings (moreover in different languages) pointing out the presence of a material connection between the influencer and the brand. Moreover, these guidelines do not mention anything about children, neither as a target audience, nor as a source (i.e., child influencers). In the EU, in 2018 the European Advertising Standards Alliance (EASA), Europe’s co-ordination point for best practice in the implementation of self-regulation, has launched its Best Practice Recommendation on Influencer Marketing^3^, as a point of reference for national guidance by self-regulatory organizations (SROs). EASA states that, “subject to local parameters, SROs may vary in their national practices and choose to go beyond what is suggested in this document to ensure that influencer marketing abides by the national advertising codes and is honest, decent and truthful and can be thus trusted by consumers” (EASA, 2018). Thus, these overarching European guidelines leave a lot of room for interpretation and implementation. In the US, the FTC provides detailed endorsement guides to instruct influencers and advertisers on what sorts of endorsements constitute advertising and where and how disclosures should be made to comply with Truth in Advertising laws in the United States (The FTC Endorsement Guides^4^). These Guides do not offer any specific information regarding children. However, the use of influencers within online platforms – such as on YouTube – necessitates additional guidelines for targeting children. The Children’s Online Privacy Protection Act or COPPA,^5^ enacted in 1998 to protect children under the age of 13, “prohibits unfair or deceptive acts or practices in connection with the collection, use, and/or disclosure of personal information from and about children on the Internet” (Electronic Code of Federal Regulations, 2019). Unboxing videos and child-influencer channels on YouTube represent one such site where young children are the audience. Recently, Google/YouTube was accused of violating COPPA by the FTC and the State of New York for collecting personal information (i.e., using personal identifiers or cookies) without parental consent and then delivering targeted advertising to the children, sometimes for products viewed within the video content (i.e., an advertisement for toys was served before the video that featured unboxing of toys; Min, 2019). Google was both claiming to toy brands such as Hasbro that their content attracted young audiences and also claiming (for regulatory purposes) that it did not have audiences under 13 so they need not worry about COPPA. In the end, Google agreed to pay a fine of $170 million and to change some of its policies and practices, including now assuming the audience to these child-targeted sites are children and halting the delivery of personalized ads there (YouTube, 2019a). Further modifications may include hiding “popularity metrics” such as the number of likes on the YouTube videos, including those of child Influencers. The measures are expected to be in place by January 2020. Some of these actions will undoubtedly influence the practice and popularity of the sites for advertisers.

Furthermore, although social media platforms include the disclosure of influencer marketing in their policies, they do not strictly enforce or control for proper disclosure. Instagram and Facebook have a branded content tool and ask users to use it when content “features or is influenced by a business partner for an exchange of value” and tag the products and brands featured. However, it is questionable whether this tagging is sufficient to help users recognize content as advertising. Furthermore, they mention that the influencers themselves are responsible for complying with legal obligations on disclosure of advertising (Facebook, 2018). YouTube also offers a “paid promotion” feature. However, including these words in their video takes some effort. Influencers should not only check the “video contains paid promotion” box in the advanced settings, they should also check the box “Help me inform viewers of paid promotion by adding a disclosure to this video. Additional disclosures for this video may be required under applicable laws” (YouTube, 2019b). This way, they actually shift the responsibility to check regulations to the influencer, without enforcing disclosure in their policy. Even YouTube Kids, YouTube’s recently launched child-friendly platform, currently has no specific rules on the proper disclosure of sponsored content. They rather merely mention that advertisements should “comply with applicable laws and regulations (including any relevant self-regulatory or industry guidelines)” (YouTube, 2019c).

Next to the protection of minor audiences, it should be noted that today influencers are often children themselves. For instance, while the FTC stresses that influencers, “shouldn’t assume legal compliance is someone else’s job” (Fair, 2019a), it is unclear to what extent minor influencers are considered responsible for the content they post, including the proper disclosure of sponsored content. Moreover, as parents or other adults such as agencies or marketers gain from children’s performances, policy makers should consider child influencers’ protection, too. For instance, it is unclear to what extent these children are protected by traditional workplace standards, such as Child Labor Laws and the California Child Actor’s Bill, which ensures that children’s earnings remain their property. Specific regulations on child labor on social media are currently lacking, while social media channels including YouTube merely inform users about the existence of labor laws, without having a clear policy (Wong, 2019).

Furthermore, ethical questions concerning these children’s privacy and possible psychological consequences of their online fame arise. Moreover, while YouTube and Instagram have an age limit of 13 years to comply with the Children’s Online Privacy Protection Act (COPPA), these age restrictions are easy to circumvent, resulting in children lying about their age and accounts created by their parents. In addition, children are often featured and even used for influencer marketing purposes in their parents’ social media outlets, such as mommy blogs, which raises questions about whether children should be protected from their parents publishing intimate or private details of their lives without consent.

To conclude, both child audiences and child influencers need protection, both through updating laws and guidelines to be applicable in the current digital times, and through translating them into clear, universal guidelines that take into account the vulnerability of minors.

## Future Research Agenda

Social media influencers’ advertising practices blur the boundaries between commercial and entertainment content. This implies that their young audience may have difficulties recognizing sponsored content such as unboxing videos as advertising, leading to subconscious persuasion (Hudders et al., 2017). Given that this persuasion tactic is relatively new, there are a number of interesting areas for future research related to influencers and children.

### Influencer Marketing Strategies

First of all, content analysis could render insights into which content strategies (child) influencers use on which platforms and how they combine different strategies and platforms. In addition, specific insights on sponsored content, including which type of brands and sectors they endorse, the branding strategies they use, the ratio between sponsored and non-sponsored posts and their use of advertising disclosures (#ad, #sponsored) could be valuable. Moreover, comments on (sponsored) posts could be analyzed (e.g., through sentiment analysis) and provide insights into children’s’ appraisal and/or criticism toward these different content strategies and the interactions between influencers and their followers. Furthermore, social network analysis could enhance our understanding of collaborations between influencers and brands and connections between influencers. Which accounts do child influencers follow (age, gender, nationality…), do they connect with other influencers and how do they engage with each other’s posts (i.e., likes, comments, views, shares…)?

Third, qualitative research could gauge for how influencers and brands feel about the strategies they use and how these may affect young children. Do they have any ethical concerns and how do they deal with those? How do they perceive their role in children’s consumer socialization? Today, influencers’ and brands’ perspectives on the practices they engage in are underexplored.

### The Impact of Influencer Marketing on Minor Audiences

To gain insights into the persuasiveness of influencers among children, scholars could examine children’s actual media use and engagement with social media platforms and influencers. Although some of the famous vloggers noted here (e. g. Ryan and Evan) have huge followers and audiences, presumably children, it is unknown if and how children under 12 years old engage with other influencers and platforms. From what age are children starting to follow vloggers? Which social media platforms are mostly used?

Additionally, little is known about how different content strategies used by influencers can differentially affect children. For instance, the prominence (subtle vs. prominent) and modality (visual vs. auditory) of a product placement can be a valuable aspect to examine. Moreover, different strategies may be exuded on different social media platforms (e.g., TikTok vs. YouTube) and also the type of brand or product (known vs. unknown, low vs. high involvement) may impact influencer marketing effects. Furthermore, there are several types of influencers – macro, micro, nano – that vary along several characteristics including number and types of followers (Ismail, 2018; Alassani and Göretz, 2019). To what extent do different kinds of influencers reach child audiences and what are the ramifications for advertising effectiveness? For example, would nano influencers look more authentic and therefore less like “slick advertising”?

Future research could probe into different kinds of influencers as important sources. Research could also examine the characteristics of these sources. To what extent do brands and consumers regard them as likeable, credible, and trustworthy? Does the strength of the parasocial interaction children feel with these influencers affect their persuasiveness? As the form and function of influencers change, scholars could try to map out conceptual similarities or differences in strategy and effects of various kinds of influencers. In addition, the possible (undesirable) longer-term effects of watching hundreds of “unboxing” videos of toys and products could be assessed. To what extent do these vloggers (unintentionally) contribute to materialism and affect children’s psychological well-being?

To answer the above research questions, it might be interesting to perform observatory or participatory research to see how children cope with social media influencers’ content in their daily life. Experimental approaches can also be valuable, although questions concerning generalizability may arise. For instance, some scholars using an experimental approach have created “fake” influencer content for stimuli in their research in an effort to enhance internal validity (Coates et al., 2019a,b). While the research has uncovered some interesting findings, it is not known to what extent the findings are generalizable to real-world influencers. Future research should work to enhance external validity as it is the very fame of the influencer that drives influence in the marketplace. On the other hand, when using existing content as stimulus material, the question may rise whether findings would be the same when using different material from different influencers. Moreover, effects may differ depending on how familiar participants are with the influencer and how they perceive him / her.

### Empowering Children to Deal With Influencer Marketing

An important issue concerning influencer marketing targeting children is how children can be empowered to cope with this contemporary advertising technique. How can we help them recognize influencer marketing and make well-informed, conscious choices?

First, an understandable advertising disclosure may help children recognize sponsored content as advertising (De Jans et al., 2018). However, up to now, research on how the inclusion of advertising disclosures may help children cope with influencer marketing has yielded mixed findings (cf. § 2.4.2.2). More research on the impact of advertising disclosures on advertising effects and source evaluations could render valuable insights that may serve as a basis for (self-)regulatory initiatives (cf. §3.4).

Second, educating children about non-traditional advertising formats, including influencer marketing could help children cope with these persuasion tactics. This could be done in a school context through an advertising literacy training (e.g., Hudders et al., 2016; Nelson, 2016; De Jans et al., 2017), which has shown to enhance children’s situational advertising literacy in some school districts in Belgium and the United States. However, there is no standard advertising literacy curriculum and it is difficult for interventions to be included within the school setting given the other curricular requirements. Moreover, the implementation of such curriculum requires specific knowledge and skills from teachers, who may have difficulties themselves in recognizing new and embedded advertising formats. Therefore, they may need to adjust or refine their own advertising literacy first (Boerman et al., 2017). In fact, children are mainly extrinsically motivated to interact with traditional learning materials as they feel under pressure from school or parents. Therefore, alternatives such as informational vlogs (e.g., De Jans et al., 2019a) or educational games (e.g., De Jans et al., 2019b) could be promising ways to include into didactic packages, as they do not take much time and can be used both in class or as a homework assignment on digital learning platforms. Moreover, as these formats are fun and entertaining, they may motivate children intrinsically to learn about new persuasion tactics (De Jans et al., 2019b). Third, training parents and encouraging them to talk with their children may present another way to instill advertising literacy. However, research suggests that the children may be watching the YouTube videos by themselves and parents may not be aware of the commercial implications of these videos (Evans et al., 2018). Moreover, it is known from literature on other embedded formats (e.g., Hudders and Cauberghe, 2018) that parental mediation is not always helpful in helping young children cope with advertising. How can parents be empowered to help their children more critically cope with influencer marketing? Research on these topics is still limited.

### Protecting Children From Influencer Marketing

Finally, although existing (self-)regulatory initiatives stress the importance of advertising being recognizable as such, especially when targeting children, a coordinated international approach on influencer marketing specifically, is non-existent today. Whereas the Federal Trade Commission in the United States requires that influencers disclose any sponsorship, the actual enforcement of this ruling is unclear in practice (Federal Trade Commission, 2017). Also in the European Union, the regulatory framework leaves room for interpretation and implementation, which led to a plethora of guidelines for disclosing sponsored content at a national level (De Jans et al., 2019a). This resulted in different types of disclosures used across influencers, platforms, and countries, which may be tricky given that the platforms transcend cultural borders and children may follow influencers from all over the world. Thus, the question arises to what extent children understand the meaning of these different disclosures and whether they help them to gain advertising literacy and enhance coping skills.

Furthermore, the question may arise whether there should be an age limit, both on targeting minors through influencer marketing as on engaging in influencer marketing activities. Concerning the latter, whereas formal guidelines to protect (child) influencers are lacking (e.g., on labor conditions and compensation) influencers are considered responsible for the content they post and are expected to comply with influencer marketing guidelines concerning the disclosure of sponsored posts (De Veirman et al., 2019). To conclude, both the practice of child influencers engaging in paid partnerships, and the targeting of children through influencer marketing raises ethical and legal concerns.

## Conclusion

The current study aimed to situate and conceptualize social media influencers as a new type of advertising source targeting children. In short, influencers can be regarded as highly popular and admired peers. While watching YouTube or scrolling through their social media apps, children are increasingly exposed to embedded advertising practices, emerging in the entertaining content social media influencers post. Influencer marketing combines the merits of eWOM and celebrity endorsement. Due to their perceived authenticity (i.e., they have no commercial interests), the (marketing) messages they spread are perceived as highly credible word of mouth, rather than as advertising. On the other hand, children look up to popular influencers who have gained a certain celebrity status and are willing to identify with them while taking on their lifestyles, attitudes and beliefs, including those on the products appearing in their social media outlets.

Prior research has shown that children indeed frequently encounter influencer marketing practices, foremost while watching YouTube (e.g., Marsh, 2016; Folkvord et al., 2019; Martínez and Olsson, 2019). The commercial content these influencers post affects children’s attitudes and behaviors, for instance their snack intake (e.g., Folkvord et al., 2019; Martínez and Olsson, 2019; Coates et al., 2019a,b; De Jans et al., 2019a). Taking into account children’s underdeveloped advertising literacy and consequently vulnerability to embedded advertising practices, such as influencer marketing, some research has searched for ways to help children recognize and cope with influencer marketing practices, for instance through the implementation of disclosures and advertising literacy interventions, such as informational vlogs (Coates et al., 2019a,b; De Jans et al., 2019a). These studies provide preliminary evidence that the implementation of a visual disclosure indeed helps children recognize influencer marketing practices as advertising, while they do not necessarily negatively impact the influencer and advertising effectiveness. Moreover, transparency is also appreciated by parents and has a positive effect on their perceptions and attitudes of both the format and the products and brands featured (Evans et al., 2018). Furthermore, three major observations arise from the (limited) literature review that aimed to gather empirical insights on the persuasiveness of influencer marketing among children. First, research primarily focuses on one particular platform, i.e., YouTube, despite the popularity of other platforms among minors and influencers accordingly (e.g., TikTok, Instagram, and Snapchat). Second, today, research on influencer marketing aimed at children is limited and mainly focusses on children between 9 and 12, while younger and thus more vulnerable children are neglected. Third, most research takes a consumer perspective and examines how children perceive influencer marketing practices, while insights on the influencer perspective and the type of content they post are lacking. A future research agenda has been set out that focuses on four potential research tracks: first, insights into influencers’ content strategies and how they perceive their role in children’s consumer socialization. Second, the impact of influencer marketing on children. Third, how to empower children to deal with influencer marketing, and fourth protecting children from influencer marketing through guidelines and regulations.

## Author Contributions

MD and LH developed the idea and outline for the article with input from MN. MD wrote the first draft of the article and MN wrote the first draft of the future research agenda. LH and MN extensively commented on the different drafts of this article. All authors have read and approved the final version of the article.

### Conflict of Interest

The authors declare that the research was conducted in the absence of any commercial or financial relationships that could be construed as a potential conflict of interest.

## Footnotes

^1^https://www.youtube.com/channel/UChGJGhZ9SOOHvBB0Y4DOO_w

^2^https://eu-pledge.eu/

^3^https://www.easa-alliance.org/sites/default/files/EASA_BEST%20PRACTICE%20RECOMMENDATION%20ON%20INFLUENCER%20MARKETING_2.pdf

^4^https://www.ftc.gov/tips-advice/business-center/guidance/ftcs-endorsement-guides-what-people-are-asking

^5^https://www.ftc.gov/enforcement/rules/rulemaking-regulatory-reform-proceedings/childrens-online-privacy-protection-rule

## References

[ref1] Abidin C. (2015). Communicative <3 Intimacies: Influencers and Perceived Interconnectedness. Ada 8. Available at: http://adanewmedia.org/2015/11/issue8-abidin/ (Accessed November 21, 2019).

[ref2] Abidin C. (2016). Visibility labour: engaging with influencers’ fashion brands and #OOTD advertorial campaigns on Instagram. Media Int. Aus. 161, 86–100. 10.1177/1329878X16665177

[ref3] Acuff D. S. Reiher R. H. (1999). What kids buy and why: The psychology of marketing to kids. New York: Free Press.

[ref4] Ahn R. Nelson M. R. (2015). Observations of food consumption in a daycare setting. Young Consum. 16, 420–437. 10.1108/YC-05-2015-00531

[ref5] Ahn R. Shen J. Gong X. Chen C. Liu W. Nelson M. R. (2019). “‘It’s time to make a change’: content analysis of food and beverage advertisements on Nickelodeon” in Annual conference of the American academy of advertising. Dallas, TX.

[ref6] Akers R. Krohn M. Lanza-Kaduce L. Radosevich M. (1979). Social learning and deviant behavior: a specific test of a general theory. Am. Sociol. Rev. 44, 636–655. 10.2307/2094592 389120

[ref7] Alassani R. Göretz J. (2019). “Product placements by micro and macro influencers on Instagram” in International conference on human-computer interaction (Cham: Springer), 251–267.

[ref8] Amos C. Holmes G. Strutton D. (2008). Exploring the relationship between celebrity endorser effects and advertising effectiveness: a quantitative synthesis of effect size. Int. J. Advert. 27, 209–234. 10.1080/02650487.2008.11073052

[ref9] Atkinson L. Nelson M. R. Rademacher M. A. (2015). A humanistic approach to understanding child consumer socialization in US homes. J. Child. Media 9, 95–112. 10.1080/17482798.2015.997106

[ref10] Bachmann G. R. John D. R. Rao A. (1993). Children’s susceptibility to peer group purchase influence: an exploratory investigation. Adv. Consum. Res. 20, 463–468.

[ref11] Bailey R. Wise K. Bolls P. (2009). How avatar customizability affects children’s arousal and subjective presence during junk food-sponsored online video games. CyberPsychol. Behav. 12, 277–283. 10.1089/cpb.2008.0292 19445632

[ref12] Bandura A. (1977). Self-efficacy: toward a unifying theory of behavioral change. Psychol. Rev. 84, 191–215. 10.1037/0033-295X.84.2.191 847061

[ref13] Bandura A. (2002). Social cognitive theory in cultural context. Appl. Psychol. Int. Rev. 51, 269–290. 10.1111/1464-0597.00092

[ref14] Bandura A. Grusec J. E. Menlove F. L. (1966). Observational learning as a function of symbolization and incentive set. Child Dev. 37, 499–506. 10.2307/1126674 4165810

[ref15] Bandura A. Ross D. Ross S. A. (1961). Transmission of aggression through imitation of aggressive models. J. Abnorm. Soc. Psychol. 63, 575–582. 10.1037/h0045925 13864605

[ref16] Bao T. Chang T.-L. S. Kim A. J. Moon S. H. (2019). The characteristics and business impact of children’s electronic word of mouth in marketing communications. Int. J. Advert. 38, 731–759. 10.1080/02650487.2018.1559558

[ref17] Basil M. D. (1996). Identification as a mediator of celebrity effects. J. Broadcast. Electron. Media 40, 478–495. 10.1080/08838159609364370

[ref18] Boerman S. C. Willemsen L. M. Van der Aa E. (2017). “This post is sponsored” effects of sponsorship disclosure on persuasion knowledge and electronic word of mouth in the context of Facebook. J. Interact. Mark. 38, 82–92. 10.1016/j.intmar.2016.12.002

[ref19] Boyd D. M. Ellison N. B. (2007). Social network sites: definition, history, and scholarship. J. Comput.-Mediat. Commun. 13, 210–230. 10.1111/j.1083-6101.2007.00393.x

[ref20] Boyland E. Harrold J. A. Dovey T. M. Halford J. C. G. (2013). Food choice and overconsumption: effect of a premium sports celebrity endorser. J. Pediatr. 163, 339–343. 10.1016/j.jpeds.2013.01.059 23490037

[ref21] Bragg M. A. Miller A. N. Elizee J. Dighe S. Elbel B. D. (2016). Popular music celebrity endorsements in food and nonalcoholic beverage marketing. Pediatrics 138, e20153977–e20153977. 10.1542/peds.2015-3977 27273712 PMC4925075

[ref22] Bragg M. A. Yanamadala S. Roberto C. A. Harris J. L. Brownell K. D. (2013). Athlete endorsements in food marketing. Pediatrics 132, 805–810. 10.1542/peds.2013-0093 24101762

[ref23] Brody G. H. Stoneman Z. (1981). Selective imitation of same-age, older and younger peer models. Child Dev. 52, 717–720. 10.2307/1129197

[ref24] Calvert S. L. (2018). Children as consumers: advertising and marketing. Futur. Child. 18, 205–234. 10.1353/foc.0.0001 21338011

[ref25] Chambers N. Kashefpakdel E. T. Rehill J. Percy C. (2018). Drawing the future: Exploring the career aspirations of primary school children from around the world. London: Education and Employers Available at: https://www.educationandemployers.org/wp-content/uploads/2018/01/Drawing-the-Future-FINAL-REPORT.pdf

[ref26] Cheung M. Y. Luo C. Sia C. L. Chen H. (2009). Credibility of electronic word-of-mouth: informational and normative determinants of on-line consumer recommendations. Int. J. Electron. Commer. 13, 9–38. 10.2753/JEC1086-4415130402

[ref27] Children’s Advertising Review Unit (2017). CARU examines YouTube channel ‘Ryan Toys Review’, recommends more prominent disclosures of Ad content. Available at: https://asrcreviews.org/caru-examines-youtube-channel-ryan-toys-review-recommends-more-prominent-disclosures-of-ad-content/ (Accessed November 21, 2019).

[ref28] Chu S. Kim Y. (2011). Determinants of consumer engagement in electronic word-of-mouth (eWOM) in social networking sites. Int. J. Advert. 30, 47–75. 10.2501/IJA-30-1-047-07

[ref29] Clement J. (2019). Most popular YouTube channels as of September 2019, ranked by number of subscribers (in millions). Available at: https://www.statista.com/statistics/277758/most-popular-youtube-channels-ranked-by-subscribers/ (Accessed November 21, 2019).

[ref30] Coates A. E. Hardman C. A. Halford J. C. G. Christiansen P. Boyland E. J. (2019a). Social media influencer marketing and children’s food intake: a randomized trial. Pediatrics 143:e20182554. 10.1542/peds.2018-2554 30833297

[ref31] Coates A. E. Hardman C. A. Halford J. C. G. Christiansen P. Boyland E. J. (2019b). The effect of influencer marketing of food and a “protective” advertising disclosure on children’s food intake. Pediatr. Obes. 14:e12540. 10.1111/ijpo.12540 31168959

[ref32] de Droog S. M. Buijzen M. Valkenburg P. M. (2012). Use a rabbit or a rhino to sell a carrot? The effect of character–product congruence on children’s liking of healthy foods. J. Health Commun. 17, 1068–1080. 10.1080/10810730.2011.650833 22650613

[ref33] de Droog S. M. Valkenburg P. M. Buijzen M. (2011). Using brand characters to promote young children’s liking of and purchase requests for fruit. J. Health Commun. 16, 79–89. 10.1080/10810730.2010.529487 21058143

[ref34] De Jans S. Cauberghe V. Hudders L. (2019a). How an advertising disclosure alerts young adolescents to sponsored vlogs: the moderating role of a peer-based advertising literacy intervention through an informational vlog. J. Advert. 47, 309–325. 10.1080/00913367.2018.1

[ref35] De Jans S. Hudders L. Cauberghe V. (2017). Advertising literacy training: the immediate versus delayed effects on children’s responses to product placement. Eur. J. Mark. 51, 2156–2174. 10.1108/EJM-08-2016-0472

[ref36] De Jans S. Hudders L. Herrewijn L. Van Geit K. (2019b). Serious games going beyond the Call of Duty: Impact of an advertising literacy mini-game platform on adolescents’ motivational outcomes through user experiences and learning outcomes. Cyberpsychol. J. Psychosocial Res. Cyberspace 13, 1–23. 10.5817/CP2019-2-3

[ref37] De Jans S. Van de Sompel D. Hudders L. Cauberghe V. (2019c). Advertising targeting young children: an overview of 10 years of research (2006–2016). Int. J. Advert. 38, 173–206. 10.1080/02650487.2017.141105639363

[ref38] De Jans S. Vanwesenbeeck I. Cauberghe V. Hudders L. Rozendaal E. van Reijmersdal E. (2018). The development and testing of a child-inspired advertising disclosure to alert children to digital and embedded advertising. J. Advert. 47, 255–269. 10.1080/00913367.2018.1463580

[ref39] De Pauw P. De Wolf R. Hudders L. Cauberghe V. (2018a). From persuasive messages to tactics: exploring children’s knowledge and judgement of new advertising formats. New Media Soc. 20, 2604–2628. 10.1177/1461444817728425

[ref40] De Pauw P. Hudders L. Cauberghe V. (2018b). Disclosing brand placement to young children. Int. J. Advert. 37, 508–525. 10.1080/02650487.2017.1335040

[ref41] De Veirman M. Cauberghe V. Hudders L. (2017). Marketing through Instagram influencers: the impact of number of followers and product divergence on brand attitude. Int. J. Advert. 36, 798–828. 10.1080/02650487.2017.1348035

[ref42] De Veirman M. De Jans S. Van den Abeele E. Hudders L. (2019). “Unraveling the power of social media influencers: a qualitative study on teenage influencers as commercial content creators on social media” in The regulation of social media influencers. eds. Goanta C. Ranchordas S. (Cheltenham, United Kingdom: Edward Elgar).

[ref43] de Vries L. Gensler S. Leeflang P. S. H. (2012). Popularity of brand posts on brand fan pages: an investigation of the effects of social media marketing. J. Interact. Mark. 26, 83–91. 10.1016/j.intmar.2012.01.003

[ref44] Devlin E. Eadie D. Stead M. Evans K. (2007). Comparative study of young people’s response to anti-smoking messages. Int. J. Advert. 26, 99–128. 10.1080/02650487.2007.11072998

[ref45] Dixon H. Scully M. Niven P. Kelly B. Chapman K. Donovan R. (2014). Effects of nutrient content claims, sports celebrity endorsements and premium offers on pre-adolescent children’s food preferences: experimental research. Pediatr. Obes. 9, e47–e57. 10.1111/j.2047-6310.2013.00169.x 23630014

[ref46] EASA (2018). EASA best practice recommendation on influencer marketing. Available at: https://www.easa-alliance.org/sites/default/files/EASA_BEST%20PRACTICE%20RECOMMENDATION%20ON%20INFLUENCER%20MARKETING_2.pdf (Accessed November 21, 2019).

[ref47] Electronic Code of Federal Regulations (2019). Title 16: commercial practices. Part 312- Children’s online privacy protection rules. Available at: https://www.ecfr.gov/cgi-bin/text-idx?SID=4939e77c77a1a1a08c1cbf905fc4b409&node=16%3A1.0.1.3.36&rgn=div5 (Accessed November 21, 2019).

[ref48] Erdogan B. Z. (1999). Celebrity endorsement: a literature review. J. Mark. Manag. 15, 291–314. 10.1362/026725799784870379

[ref49] EU Pledge (2019). About the EU pledge. Available at: https://eu-pledge.eu/about-the-eu-pledge/ (Accessed November 21, 2019).

[ref50] Evans N. J. Hoy M. G. Childers C. C. (2018). Parenting “YouTube natives”: the impact of pre-roll advertising and text disclosures on parental responses to sponsored child influencer videos. J. Advert. 47, 326–346. 10.1080/00913367.2018.1544952

[ref51] Facebook (2018). Branded content policies. Available at: https://www.facebook.com/policies/brandedcontent/ (Accessed November 21, 2019).

[ref52] Fair L. (2019a). FTC-FDA warning letters: Influential to influencers and marketers. Available at: https://www.ftc.gov/news-events/blogs/business-blog/2019/06/ftc-fda-warning-letters-influential-influencers-marketers (Accessed November 21, 2019).

[ref53] Fair L. (2019b). Great American Fake-Off? FTC cases challenge bogus influencer metrics and fake reviews. Available at: https://www.ftc.gov/news-events/blogs/business-blog/2019/10/great-american-fake-ftc-cases-challenge-bogus-influencer (Accessed November 21, 2019).

[ref54] Federal Trade Commission (2017). The FTC’s endorsement guides: what people are asking. Available at: https://www.ftc.gov/tips-advice/business-center/guidance/ftcs-endorsement-guides-what-people-are-asking (Accessed November 21, 2019).

[ref55] Flanagin A. J. Metzger M. J. (2007). The role of site features, user attributes, and information verification behaviors on the perceived credibility of web-based information. New Media Soc. 9, 319–342. 10.1177/1461444807075015

[ref56] Flanders J. P. (1968). A review of research on imitative behavior. Psychol. Bull. 69, 316–337. 10.1037/h0025721 4873055

[ref57] Folkvord F. Bevelander K. E. Rozendaal E. Hermans R. C. J. (2019). Children’s bonding with popular YouTube vloggers and their attitudes towards brand and product endorsements in vlogs: an explorative study. Young Consum. 20. 10.1108/YC-12-2018-0896

[ref58] Friestad M. Wright P. (1994). The persuasion knowledge model: how people cope with persuasion attempts. J. Consum. Res. 21, 1–31. 10.1086/209380

[ref59] Gannon V. Prothero A. (2018). Beauty bloggers and YouTubers as a community of practice. J. Mark. Manag. 34, 592–619. 10.1080/0267257X.2018.1482941

[ref60] Gilbert D. T. Malone P. S. (1995). The correspondence bias. Psychol. Bull. 117, 21–38. 10.1037/0033-2909.117.1.21 7870861

[ref61] Guttmann A. (2019). Spending on advertising to children worldwide from 2012 to 2021, by format (in billion U.S. dollars). Available at: https://www.statista.com/statistics/750865/kids-advertising-spending-worldwide/ (Accessed November 21, 2019).

[ref62] Hebden L. King L. Kelly B. (2011). Art of persuasion: an analysis of techniques used to market foods to children. J. Paediatr. Child Health 47, 776–782. 10.1111/j.1440-1754.2011.02025.x 21707822

[ref63] Hoffner C. (2008). “Parasocial and online social relationships” in The handbook of children, media and development. eds. Calvert S. L. Wilson B. J. (Blackwell-Wiley: Hoboken).

[ref64] Hoffner C. Buchanan M. (2005). Young adults’ wishful identification with television characters: the role of perceived similarity and character attributes. Media Psychol. 7, 325–351. 10.1207/S1532785XMEP0704_2

[ref65] Horton D. Wohl R. R. (1956). Mass communication and Para-social interaction: observations on intimacy at a distance. Psychiatry 19, 215–229. 13359569 10.1080/00332747.1956.11023049

[ref66] Hovland C. I. Janis I. L. Kelley H. H. (1953). Communication and persuasion; psychological studies of opinion change. New Haven, CT, US: Yale University Press.

[ref67] Hsu T. (2019). Popular YouTube toy review channel accused of blurring lines for ads. New York: The New York Times Available at: https://www.nytimes.com/2019/09/04/business/media/ryan-toysreview-youtube-ad-income.html

[ref601] Hudders L. Cauberghe V. (2018). The mediating role of advertising literacy and the moderating influence of parental mediation on how children of different ages react to brand placements. J. Consum. Behav. 17, 197–210. 10.1002/cb.1704

[ref68] Hudders L. Cauberghe V. Panic K. (2016). How advertising literacy training affect children’s responses to television commercials versus advergames. Int. J. Advert. 35, 909–931. 10.1080/02650487.2015.1090045

[ref69] Hudders L. De Pauw P. Cauberghe V. Panic K. Zarouali B. Rozendaal E. (2017). Shedding new light on how advertising literacy can affect children’s processing of embedded advertising formats: a future research agenda. J. Advert. 46, 333–349. 10.1080/00913367.2016.1269303

[ref70] Ismail K. (2018). Social media influencers: mega, macro, micro or nano. Available at: https://www.cmswire.com/digital-marketing/social-media-influencers-mega-macro-micro-or-nano/ (Accessed November 21, 2019).

[ref71] Jain V. Roy S. Daswani A. Sudha M. (2011). What really works for teenagers: human or fictional celebrity? Young Consum. 12, 171–183. 10.1108/17473611111141623

[ref72] John D. R. (1999). Consumer socialization of children: a retrospective look at twenty-five years of research. J. Consum. Res. 26, 183–213. 10.1086/209559

[ref74] Kahle L. Homer P. (1985). Physical attractiveness of the celebrity endorser: a social adaptation perspective. J. Consum. Res. 11, 954–961. 10.1086/209029

[ref75] Kamins M. A. (1989). Celebrity and non-celebrity advertising in a two-sided context. J. Advert. Res. 29, 34–42.

[ref76] Kamins M. A. (1990). An investigation into the “match-up” hypothesis in celebrity advertising: when beauty may be only skin deep. J. Advert. 19, 4–13. 10.1080/00913367.1990.10673175

[ref77] Kamins M. A. Brand M. J. Hoeke S. A. Moe J. C. (1989). Two-sided versus one-sided celebrity endorsements: the impact on advertising effectiveness and credibility. J. Advert. 18, 4–10. 10.1080/00913367.1989.10673146

[ref78] Kelman H. C. (1961). Processes of opinion change. Public Opin. Q. 25, 57–78. 10.1086/266996

[ref79] Kelman H. C. (2006). “Interests, relationships, identities: three central issues for individuals and groups in negotiating their social environment” in Annual review of psychology. eds. Fiske S. T. Kazdin A. E. Schacter D. L., Vol. 57 (Palo Alto, CA: Annual Reviews). 10.1146/annurev.psych.57.102904.190156 16318587

[ref80] Khamis S. Ang L. Welling R. (2017). Self-branding, ‘micro-celebrity’ and the rise of social media influencers. Celebrity Stud. 8, 191–208. 10.1080/19392397.2016.1218292

[ref81] Knoll J. (2016). Advertising in social media: a review of empirical evidence. Int. J. Advert. 35, 266–300. 10.1080/02650487.2015.1021898

[ref82] Knoll J. Matthes J. (2017). The effectiveness of celebrity endorsements: a meta-analysis. J. Acad. Mark. Sci. 45, 55–75. 10.1007/s11747-016-0503-8

[ref83] Kraak V. I. Story M. (2015). Influence of food companies’ brand mascots and entertainment companies’ cartoon media characters on children’s diet and health: a systematic review and research needs. Obes. Rev. 16, 107–126. 10.1111/obr.12237 25516352 PMC4359675

[ref84] Lagomarsino M. Suggs S. L. (2018). Choosing imagery in advertising healthy food to children: are cartoons the most effective visual strategy? J. Advert. Res. 59, 487–498. 10.2501/JAR-2018-003

[ref85] Lapierre M. A. Vaala S. E. Linebarger D. L. (2011). Influence of licensed spokescharacters and health cues on children’s ratings of cereal taste. Arch. Pediatr. Adolesc. Med. 165, 229–234. 10.1001/archpediatrics.2010.300 21383272

[ref86] Lasswell H. D. (1948). Power and personality. New York: Norton.

[ref600] Lee J. E. Watkins B. (2016). YouTube vloggers’ influence on consumer luxury brand perceptions and intentions. J. Bus. Res. 69, 5753–5760. 10.1016/j.jbusres.2016.04.171

[ref87] Lloyd B. T. (2002). A conceptual framework for examining adolescent identity, media influence, and social development. Rev. Gen. Psychol. 6, 73–91. 10.1037/1089-2680.6.1.73

[ref88] Lou C. Yuan S. (2019). Influencer marketing: how message value and credibility affect consumer trust of branded content on social media. J. Interact. Advert. 19, 58–73. 10.1080/15252019.2018.1533501

[ref89] Lyons B. Henderson K. (2005). Opinion leadership in a computer-mediated environment. J. Consum. Behav. 4, 319–329. 10.1002/cb.22

[ref90] Mangleburg T. F. Doney P. M. Bristol T. (2004). Shopping with friends, and teen’s susceptibility to peer influence. J. Retail. 80, 101–116. 10.1016/j.jretai.2004.04.005

[ref91] Marsh J. (2016). ‘Unboxing’ videos: co-construction of the child as cyberflâneur. Discourse 37, 369–380. 10.1080/01596306.2015.1041457

[ref92] Martínez C. Olsson T. (2019). Making sense of YouTubers: how Swedish children construct and negotiate the YouTuber Misslisibell as a girl celebrity. J. Child. Media 13, 36–52. 10.1080/17482798.2018.1517656

[ref93] McCracken G. (1989). Who is the celebrity endorser? Cultural foundations of the endorsement process. J. Consum. Res. 16, 310–321. 10.1086/209217

[ref94] McGuire W. J. (1985). “Attitudes and attitude change” in Handbook in social psychology. eds. Gardner L. Aronson E. (New York: Random House).

[ref95] McNeal J. U. (2007). On becoming a consumer: Development of consumer behavior patterns in childhood. Woburn, MA, US: Butterworth-Heinemann.

[ref96] Min S. (2019). Google to pay $170 million for violating kids’ privacy on YouTube. Available at: https://www.cbsnews.com/news/ftc-fines-google-170-million-for-violating-childrens-privacy-on-youtube/ (Accessed November 21, 2019).

[ref97] Naderer B. Matthes J. Spielvogel I. (2019). How brands appear in children’s movies. A systematic content analysis of the past 25 years. Int. J. Advert. 38, 237–257. 10.1080/02650487.2017.1410000

[ref98] Naderer B. Matthes J. Zeller P. (2018). Placing snacks in children’s movies: cognitive, evaluative, and conative effects of product placements with character product interaction. Int. J. Advert. 37, 852–870. 10.1080/02650487.2017.1348034

[ref99] Nairn A. Fine C. (2008). Who’s messing with my mind? Int. J. Advert. 27, 447–470. 10.2501/S0265048708080062

[ref100] Neeley S. M. Schumann D. W. (2004). Using animated spokes-characters in advertising to young children. J. Advert. 33, 7–23. 10.1080/00913367.2004.10639166

[ref101] Nelson M. R. (2016). Developing persuasion knowledge by teaching advertising literacy in primary school. J. Advert. 45, 169–182. 10.1080/00913367.2015.1107871

[ref102] Nelson M. R. Duff B. R. Ahn R. (2015). Visual perceptions of snack packages among preschool children. Young Consum. 16, 385–406. 10.1108/YC-02-2015-00507

[ref103] Nicoll B. Nansen B. (2018). Mimetic production in YouTube toy unboxing videos. Soc. Media Society 4, 1–12. 10.1177/2056305118790761

[ref104] Novacic I. (2019). “It’s kinda crazy”: Kid influencers make big money on social media, and few rules apply. CBS News? Available at: https://cbsn.ws/2ZhQ8sf (Accessed November 21, 2019).

[ref105] O’Connell L. (2019). Consumer spending on children’s licensed merchandise products in the United States in quarter four of 2017 and 2018 (in billion U.S. dollars). Available at: https://www.statista.com/statistics/1034219/consumer-spending-on-kids-licensed-merchandise-us/ (Accessed November 21, 2019).

[ref106] O’Sullivan E. (2003). Bringing a perspective of transformative learning to globalized consumption. J. Consumer Stud. 27, 326–330. 10.1046/j.1470-6431.2003.00327.x

[ref107] Ofcom (2019). Children and parents: media use and attitudes report 2018. Available at: https://www.ofcom.org.uk/__data/assets/pdf_file/0024/134907/Children-and-Parents-Media-Use-and-Attitudes-2018.pdf (Accessed November 21, 2019).

[ref108] Ogle A. D. Graham D. J. Lucas-Thompson R. G. Roberto C. A. (2017). Influence of cartoon media characters on children’s attention to and preference for food and beverage products. J. Acad. Nutr. Diet. 117, 265–270. 10.1016/j.jand.2016.08.012 27793520 PMC5574189

[ref109] Ohanian R. (1991). The impact of celebrity spokespersons’ perceived image on consumers’ intention to purchase. J. Advert. Res. 31, 46–54.

[ref110] Pew Research Center (2018). Many turn to YouTube for children’s content, news, how-to lessons. Available at: https://www.pewinternet.org/2018/11/07/many-turn-to-youtube-for-childrens-content-news-how-to-lessons/ (Accessed November 21, 2019).

[ref111] Phelps J. E. Lewis R. Mobilio L. Perry D. Raman N. (2004). Viral marketing or electronic word-of-mouth advertising: examining consumer responses and motivations to pass along email. J. Advert. Res. 44, 333–348. 10.1017/S0021849904040371

[ref112] Pilgrim K. Bohnet-Joschko S. (2019). Selling health and happiness how influencers communicate on Instagram about dieting and exercise: mixed methods research. BMC Public Health 19:1054. 10.1186/s12889-019-7387-8 31387563 PMC6683418

[ref113] Power S. Smith K. (2017). ‘Heroes’ and ‘villains’ in the lives of children and young people. Discourse Stud. Cult. Polit. Edu. 38, 590–602. 10.1080/01596306.2015.1129311

[ref114] Read B. (2011). Britney, Beyoncé, and me – primary school girls’ role models and constructions of the ‘popular’ girl. Gend. Educ. 23, 1–13. 10.1080/09540251003674089

[ref115] Robehmed N. Berg M. (2018). Highest-Paid YouTube Stars 2018: Markiplier, Jake Paul, PewDiePie and More. Available at: https://www.forbes.com/sites/natalierobehmed/2018/12/03/highest-paid-youtube-stars-2018-markiplier-jake-paul-pewdiepie-and-more/#563480c2909a (Accessed November 21, 2019).

[ref116] Ross R. P. Campbell T. Wright J. C. Huston A. C. Rice M. L. Turk P. (1984). When celebrities talk, children listen: an experimental analysis of children’s responses to TV ads with celebrity endorsement. J. Appl. Dev. Psychol. 5, 185–202. 10.1016/0193-3973(84)90017-0

[ref117] Rozendaal E. Buijzen M. Valkenburg P. (2011). Children’s understanding of advertisers’ persuasive tactics. Int. J. Advert. 30, 329–350. 10.2501/IJA-30-2-329-350

[ref118] Rubin A. M. Perse E. M. Powell R. A. (1985). Loneliness, parasocial interaction, and local television news viewing. Hum. Commun. Res. 12, 155–180. 10.1111/j.1468-2958.1985.tb00071.x

[ref119] Russell C. A. (1998). “Toward a framework of product placement: theoretical propositions” in NA - advances in consumer research. Vol. 25 eds. Alba J. W. Hutchinson J. W. (Provo, UT: Association for Consumer Research).

[ref120] Schouten A. P. Janssen L. Verspaget M. (2019). Celebrity vs. influencer endorsements in advertising: the role of identification, credibility, and product-endorser fit. Int. J. Advert.. Advance Online Publication. 10.1080/02650487.2019.1634898

[ref121] Smits T. Vandebosch H. Neyens E. Boyland E. (2015). The persuasiveness of child-targeted endorsement strategies: a systematic review. Commun. Yearbook 39, 311–338. 10.1080/23808985.2015.11679179

[ref122] Stafford M. R. Stafford T. F. Day E. (2002). A contingency approach: the effects of spokesperson type and service type on service advertising perceptions. J. Advert. 31, 17–35. 10.1080/00913367.2002.10673664

[ref123] Stern B. (1994). A revised model for advertising: multiple dimensions of the source, the message, andthe recipient. J. Advert. 23, 5–16. 10.1080/00913367.1994.10673438

[ref124] Sternthal B. Phillips L. Dholakia R. (1978). The persuasive effect of source credibility: a situational analysis. Public Opin. Q. 42, 285–314. 10.1086/268454 10297222

[ref125] Till B. D. Shimp T. A. (1998). Endorsers in advertising: the case of negative celebrity information. J. Advert. 27, 67–82. 10.1080/00913367.1998.10673543

[ref126] Tsay-Vogel M. Schwartz M. L. (2014). Theorizing parasocial interactions based on authenticity: the development of a media figure classification scheme. Psychol. Pop. Media Cult. 3, 66–78. 10.1037/a0034615

[ref127] Uzunoglu E. Kip M. S. (2014). Brand communication through digital influencers: leveraging blogger engagement. Int. J. Inf. Manag. 34, 592–602. 10.1016/j.ijinfomgt.2014.04.007

[ref128] van Dam S. van Reijmersdal E. (2019). Insights in adolescents’ advertising literacy, perceptions and responses regarding sponsored influencer videos and disclosures. Cyberpsychol. J. Psychosoc. Res. Cyberspace 13. 10.5817/CP2019-2-2

[ref129] Van de Sompel D. Vermeir I. (2016). The influence of source attractiveness on self-perception and advertising effectiveness for 6- to 7-year-old children. Int. J. Consum. Stud. 40, 575–582. 10.1111/ijcs.12302

[ref130] van Noort G. Antheunis M. L. van Reijmersdal E. A. (2012). Social connections and the persuasiveness of viral campaigns in social network sites: persuasive intent as the underlying mechanism. J. Mark. Commun. 18, 39–53. 10.1080/13527266.2011.620764

[ref131] Watson A. (2019). Children and media in the U.S. – statistics & facts. Available at: https://www.statista.com/topics/3980/children-and-media-in-the-us/ (Accessed November 21, 2019).

[ref132] Weiss G. (2018). YouTube To Net $3.4 Billion In U.S. Ad Revenues This Year (Study). Available at: https://www.tubefilter.com/2018/10/09/youtube-3-billion-us-ad-revenues/ (Accessed November 21, 2019).

[ref133] Wilson E. J. Sherrell D. L. (1993). Source effects in communication and persuasion research: a meta-analysis of effect size. J. Acad. Mark. Sci. 21, 101–112. 10.1007/BF02894421

[ref134] Wojdynski B. W. Evans N. J. (2016). Going native: effects of disclosure position and language on the recognition and evaluation of online native advertising. J. Advert. 45, 157–168. 10.1080/00913367.2015.1115380

[ref135] Wong J. C. (2019). ‘It’s not play if you’re making money’: how Instagram and YouTube disrupted child labor laws. Available at: https://www.theguardian.com/media/2019/apr/24/its-not-play-if-youre-making-money-how-instagram-and-youtube-disrupted-child-labor-laws (Accessed November 21, 2019).

[ref136] Yang F. X. (2017). Effects of restaurant satisfaction and knowledge sharing motivation on eWOM intentions: the moderating role of technology acceptance factors. J. Hosp. Tour. Res. 41, 93–127. 10.1177/1096348013515918

[ref137] YouTube (2019a). An update on kids and data protection on YouTube. Available at: https://youtube.googleblog.com/2019/09/an-update-on-kids.html (Accessed November 21, 2019).

[ref138] YouTube (2019b). Paid product placements and endorsements. Available at: https://support.google.com/youtube/answer/154235?hl=en (Accessed November 21, 2019).

[ref139] YouTube (2019c). Advertising on YouTube Kids. Available at: https://support.google.com/youtube/answer/6168681?hl=en (Accessed November 21, 2019).

